# The technical efficiency of maternal and child health hospitals in China: a case study of Hubei Province

**DOI:** 10.1186/s12978-022-01386-x

**Published:** 2022-03-31

**Authors:** Dongdong Jiang, Xinliang Liu, Yan Chen, Jinwei Hao, Hao Huang, Qian Huang, Qinghua Chen, Quan Wang, Hao Li

**Affiliations:** 1 School of Public Health/Global Health Institute, Wuhan University, Wuhan, China; 2 School of Health Sciences, Faculty of Biology, Medicine and Health, The University of Manchester, Manchester, UK; 3 School of Public Health, Zhejiang University, Hangzhou, China; 4 Department of Maternal and Child Health, Health Commission of Hubei Province, Wuhan, China

**Keywords:** Technical efficiency, Secondary maternal and child health hospitals, Resource allocation, Bootstrap-Data Envelopment Analysis

## Abstract

**Background:**

Maternal and child health (MCH) hospitals play an essential role in providing MCH services in China, while the supply has become increasingly challenging in the past decade, especially among secondary MCH hospitals. In this study we aimed to evaluate the technical efficiency (TE) of secondary MCH hospitals in Hubei province (China) to generate evidence-based decision-making for efficiency improvement.

**Methods:**

The data were collected from the Department of Maternal and Child Health of Health Commission of Hubei Province in 2019. A total of 59 out of 74 secondary MCH hospitals were included as our study sample. Four input indicators (number of health professionals, number of beds, number of equipment with value greater than 10,000 RMB Yuan, building area for hospital operations) and three output indicators (number of total diagnostic patients, number of discharged patients, and number of birth deliveries) were selected based on previous literature. TE scores of the sample hospitals were estimated by using Bootstrap-Data Envelopment Analysis (Bootstrap-DEA).

**Results:**

After bias-correction with Bootstrap-DEA model, the average TE score of the MCH hospitals was 0.673. 20 (33.89%) MCH hospitals had TE scores below the average. No MCH hospitals achieved excellent efficiency; 16 (27.11%) MCH hospitals reached good efficiency; and 26 (44.06%) MCH hospitals fell into poor and failing efficiency. Besides, 17 MCH hospitals had TE scores of 1 before bias-corrections, while none of them reached 1 after bias correction.

**Conclusions:**

Significant capacity variations existed among the secondary MCH hospitals in terms of input and output indicators and their overall TE was low in Hubei of China. For better improvement, the secondary MCH hospitals in Hubei need to improve their internal management and strengthen the construction of their information systems. A series of policy supports are needed from the health and insurance administrations to optimize health resources. Third-party performance evaluation can be piloted to improve efficiency and overall performance of the MCH hospitals. The policy recommendations we raise for MCH hospitals in Hubei can be worth learning for some low- and middle- income countries.

**Supplementary Information:**

The online version contains supplementary material available at 10.1186/s12978-022-01386-x.

## Plain language summary

Efficient operation is key for maternal and child health (MCH) hospitals to provide services to meet the increasing healthcare demands in China. Traditional Data Envelopment Analysis (DEA) models fail to generate efficiency scores excluding the impact of the environmental and random factors, which may mislead evidenced decision-making. In this study, we introduced the Bootstrap-DEA model to adjust the bias. The bias-corrected technical efficiency (TE) scores were estimated based on 59 secondary MCH hospitals in Hubei province of China in 2019. We found that: (1) significant capacity variations existed among the hospitals in terms of input and output indcators; (2) the bias-corrected TE scores were all lower than those without bias-correction; and (3) over 1/3 MCH hospitals had an efficiency score lower than the average (0.673). Although China has made outstanding achievements in MCH at the national level, our findings further indicated aspects to be addressed at the provincial and local levels. It is suggested that actions such as health resources optimization, information systems capacity building, MCH hospital internal management, benchmarking and performance evaluation, etc. could be possible directions for further implementation. In addition, the suggestions we proposed for MCH hospitals in Hubei can also have some policy implications for some low- and middle- income countries.

## Background

China has achieved the Sustainable Development Goals (SDGs) 3.1 and 3.2 on maternal and children health (MCH) in advance. The data of the neonatal mortality, under-5 mortality, and maternal mortality in China were 3.40/1000, 7.50/1000, 16.90/1,000,000 in 2020 [1], respectively, while the counterpart threshold goals set by SDGs are 12/1000, 25/1000, 70/100,000 in 2030. Because the number of women accounted for 17.86% of the global female population and the number of children accounted for 12.64% of the global child population in 2019 [2], China has made significant contributions to global MCH by achieving its domestic SDGs in advance. However, many low- and middle- income countries (LMICs) and regions still have problems and challenges in providing MCH services [3], and these countries may benefit from learning China’s experience. In China’s way to achieving the SDGs, MCH hospitals have played a crucial role by providing efficient services with quality.

However, the number of MCH hospitals in China is less than one-sixth of the number of general hospitals [4], which means that the development of MCH hospitals is still lagged behind compared to general hospitals in their capacity. In China, although MCH service provision at the national level has performed better compared with most of the other countries [5], disparities across regions still exist [6], calling for more research at regional levels. Moreover, China has implemented the universal two-child policy since 2015, and the governments at all levels encouraged each couple to raise a maximum of three children in 2021[7]. The demand for MCH services has been increasing, which calls for optimization of health resources allocation. The advantageous health resources in Hubei province, for example, mainly concentrate in big tertiary MCH hospitals [8], and this may result in not only a surplus of resources in these hospitals, but also insufficient resources for lower levels of MCH hospitals, while secondary MCH hospitals are core to meet the increasing demands at the district and county levels. However, secondary MCH hospitals’ efficiency has yet to be further studied for resource optimization and improvement.

In terms of efficiency measurement in healthcare institutions, parametric and non-parametric methods have been widely recognized and applied worldwide. Like stochastic frontier analysis (SFA), the parametric method has less been used in complex contexts because it only applies to single output [9]. However, the non-parametric method like Data Envelopment Analysis (DEA) applies to relative efficiency analysis with multiple inputs and outputs. Several classical DEA models are frequently-used worldwide, including Charnes, Cooper, and Rhodes (CCR, 1978) [10], Banker, Charnes, and Cooper (BCC, 1982) [11], and Malmquist-DEA [12, 13], etc. However, due to environmental and random factors, the efficiency scores shall fall into a fluctuating range [9]. To generate more reliable estimation results, Bootstrap-DEA was introduced by Simar and Wilson (1998) to correct the bias of efficiency scores and to calculate lower and upper bounds of confidence intervals [14, 15]. Moreover, according to Fare et al. (1994), efficiency consists of allocative efficiency (AE) and technical efficiency (TE) [12]. Because of the difficulty to collect price information of input indicators required by AE measurement, many studies focused on the measurement of TE, which only requires inputs and outputs information measured in volume [16].

In China, the studies on efficiency evaluation of secondary MCH hospitals are limited [8, 17–19]. Most of them are still based on the classical CCR and BCC models without bias correction of efficiency scores. Therefore, the purpose of this study was to introduce the Bootstrap-DEA model to evaluate the TE of secondary MCH hospitals in Hubei province, China with policy implications.

## Methods

### Context and sample

Hubei province is located in the central region of China, with a population of 59.27 million in 2019, which is close to the population of Italy (60.48 million) [20]. The gross domestic product of Hubei reached 655.62 billion dollars, which surpassed Poland’s (595.86 billion dollars, ranking top No.21 in the world) [20].

MCH hospitals in China are medical institutions specifically designed to offer MCH services, including primary and public health services for women and children. MCH hospitals are divided into provincial, prefecture, and county (district) levels based on their affiliated administrative divisions. Further, MCH hospitals are divided into three levels, with the third level representing the highest standard. MCH hospitals at different levels vary in capacity, department setting, medical staff, etc. For example, it is stipulated that the number of inpatient beds for a second-level MCH hospital shall be between 20 and 49, with not less than 40 health professionals. In contrast, the number of inpatient beds for a tertiary MCH hospital shall be at least 50, with at least 60 health professionals. In 2019, altogether there were 106 MCH hospitals in Hubei province.

### Selection of input and output indicators

Based on our previous hospital efficiency research using DEA models, only direct volume input and output indicators shall be included, and monetary and ratio indicators shall be excluded [9, 16, 21]. Therefore, number of health professionals, number of beds, number of equipment with value greater than 10,000 RMB Yuan, building area for hospital operations were selected as input indicators, while number of total diagnostic patients and number of discharged patients were selected as output indicators. In addition, to the best of our knowledge, few Chinese literature have included MCH featured indicators into the output indicators [18, 22, 23]. Therefore, we included the indicator “number of birth deliveries” into the output indicators among all others.

### Bootstrap-DEA model

The principle of Bootstrap-DEA is to simulate the data generating process by repeated sampling. Since the simulated dataset is approximately equal to the original one, the sampling distributions and standard deviations of the simulated dataset are close to the original one. Moreover, the Bootstrap-DEA model can obtain simulated efficiency scores by setting the number of repeated sampling, thereby generating the bias-corrected efficiency scores and confidence intervals at α = 0.05 level. In this way, the efficiency scores will be more accurate.

The formulas on how to estimate the TE with the Bootstrap-DEA model are as follows [14, 15]:

$${\text{Bias}}\left( {\hat{\theta }_{k} } \right) = E\left( {\hat{\theta }_{k} } \right) - \hat{\theta }_{k}$$

$${\text{Bias}}\left( {\hat{\theta }_{k} } \right) = B^{ - 1} \mathop \sum \limits_{b = 1}^{B} \left( {\hat{\theta }_{kb}^{*} } \right) - \hat{\theta }_{k}$$

The bias corrected efficiency score can be attained by the formula below:

$$\tilde{\theta }_{k} = \hat{\theta }_{k} - Bias\left( {\hat{\theta }_{k} } \right) = 2\hat{\theta }_{k} - B^{ - 1} \mathop \sum \limits_{b = 1}^{B} \left( {\hat{\theta }_{kb}^{*} } \right)$$

The confidential interval at α confidence level can be expressed as follows:

$$P_{r} \left( { - \hat{b}_{r} \le \hat{\theta }_{kb}^{*} - \hat{\theta }_{k} \le - \hat{a}_{a} } \right) = 1 - \alpha$$

$$P_{r} \left( { - \hat{b}_{a} \le \hat{\theta }_{k}^{*} - \hat{\theta }_{k} \le - \hat{a}_{a} } \right) = 1 - \alpha$$

$$\hat{\theta }_{k} + \hat{a}_{a} \le \hat{\theta }_{k} \le \hat{\theta }_{k} + \hat{b}_{a}$$

### Data collection and processing

The data were collected from the Department of Maternal and Child Health of the Health Commission of Hubei Province (HCHBP) in 2019. Seventy-four secondary MCH hospitals were selected in this study. Among them, 15 secondary MCH hospitals reported incomplete data in some key indicators (such as the number of health professionals and the number of birth deliveries). Therefore, only 59 MCH hospitals had complete data and can meet our requirement for further analysis.

### Data analysis

Descriptive information was analyzed by using R software (version 3.2.1.) and FEAR package was used to estimate the TE scores of MCH hospitals with Bootstrap-DEA [24, 25]. Efficiency scores before bias-corrections would return to Farrell scores [26]. After the Bootstrap (2000 times of repeated sampling, the α of confidence intervals taken as 0.05), the efficiency scores, bias, and lower and upper bound based on Shephard’s output distance functions would be returned [27]. In order to support policy decision-making, benchmarking and ranking were applied. All the DMUs will be classified into 5 groups (excellent, good, average, poor, and failing) [9, 28]. In order to have better visual reporting, different colors were applied in relation to different efficiency levels of the DMUs, with dark green to indicate excellent efficiency (scores ∈ [0.900, 1.000]), green to represent good efficiency (scores ∈ [0.800, 0.900]), yellow to describe average efficiency (scores ∈ [0.700, 0.800]), brown to show poor efficiency (scores ∈ [0.600, 0.700]), and red to present failing efficiency ( (scores ∈ [0.000, 0.600])). Moreover, due to privacy concerns, all information of MCH hospitals was set as anonymous, and each hospital was assigned a sequence number orderly from 1 to 59. Rankings were sorted by descending bias-corrected efficiency scores.

## Results

### Descriptive statistics

As described in Table 1, there was a significantly wide capacity variation among all indicators in different secondary MCH hospitals. In terms of each of the input indicators, the number of secondary MCH hospitals below average was 35 (59.32%), 36 (61.02%), 31 (52.54%), and 32 (54.23%), respectively. Regarding the output indicators, the corresponding numberof secondary MCH hospitals below average were 33 (55.93%), 33 (55.93%), and 35 (59.32%), respectively.

**Table 1:** Descriptive information of input–output indicators of the 59 MCH hospitals

	Mean	SD	Minimum	Maximum
Input indicators				
Number of health professionals	193.73	93.81	56	560
Number of beds	211.46	112.27	30	450
Number of equipment with value greater than 10,000 RMB Yuan	329.80	185.54	54	1054
Building area for hospital operation (m^2^)	10,382.96	7973.35	2414	44,432
Output indicators				
Number of total diagnostic patients	121,325.51	74,143.59	17,238	299,505
Number of discharged patients	5287.17	4174.24	75	22,348
Number of birth deliveries	5485.98	3308.56	786	19,836

*SD* standard deviation

### TE scores and ranking before and after bias-corrections of the MCH hospitals

As shown in Table 2, all the bias-corrected TE scores were lower than those before bias-correction. The biggest bias before and after bias-corrections was 0.327 (DMU44). The highest bias-corrected TE score was 0.885 (DMU12), while the lowest was 0.217 (DMU15). The geometric mean of samples before bias-correction was 0.789, and the geometric mean after bias-correction was 0.673. Seventeen secondary MCH hospitals had TE scores of 1 before bias-corrections, while none of them reached 1 after bias correction. In particular, one MCH hospital (DMU44) even ranked at 40th after bias-correction.

**Table 2:** Efficiency scores and rankings before and after bias correction of MCH hospitals

DMU	Before bias-correction	After bias-correction	Bias	Lower bound	Upper bound	Ranking orders [After (Before)]
DMU 12	0.995	0.885	0.110	0.821	0.987	1 (18)
DMU 11	0.977	0.877	0.101	0.828	0.970	2 (20)
DMU 4	1.000	0.875	0.125	0.831	0.990	3 (1)
DMU 42	0.971	0.853	0.118	0.789	0.965	4 (21)
DMU 28	1.000	0.844	0.156	0.796	0.991	5 (1)
DMU 59	1.000	0.844	0.156	0.801	0.990	6 (1)
…	…	…	…	…	…	…
DMU 52	0.930	0.817	0.113	0.767	0.920	13 (24)
DMU 36	0.946	0.813	0.133	0.737	0.942	14 (22)
DMU 46	0.881	0.813	0.069	0.781	0.874	15 (27)
DMU 43	0.925	0.806	0.119	0.755	0.917	16 (25)
DMU 57	0.889	0.785	0.104	0.744	0.882	17 (26)
DMU 7	0.825	0.767	0.058	0.730	0.818	18 (31)
…	…	…	…	…	…	…
DMU 45	1.000	0.733	0.267	0.680	0.989	25 (1)
DMU 6	1.000	0.730	0.270	0.711	0.990	26 (1)
DMU 50	1.000	0.730	0.270	0.711	0.991	27 (1)
DMU 54	0.815	0.724	0.091	0.680	0.809	28 (32)
DMU 56	0.771	0.722	0.050	0.696	0.764	29 (38)
DMU 23	0.780	0.715	0.065	0.688	0.773	30 (36)
…	…	…	…	…	…	…
DMU 47	0.783	0.692	0.091	0.655	0.778	37 (35)
DMU 2	0.768	0.679	0.089	0.646	0.761	38 (39)
DMU 1	1.000	0.676	0.324	0.655	0.992	39 (1)
DMU 44	1.000	0.673	0.327	0.648	0.992	40 (1)
DMU 8	0.711	0.668	0.043	0.636	0.706	41 (45)
DMU 34	0.756	0.663	0.093	0.627	0.749	42 (41)
…	…	…	…	…	…	…
DMU 55	0.557	0.477	0.081	0.430	0.553	54 (53)
DMU 25	0.464	0.433	0.032	0.415	0.460	55 (56)
DMU 17	0.464	0.429	0.034	0.409	0.459	56 (57)
DMU 14	0.469	0.425	0.044	0.403	0.465	57 (55)
DMU 18	0.419	0.378	0.040	0.360	0.414	58 (58)
DMU 15	0.239	0.217	0.022	0.207	0.236	59 (59)
G mean	0.789	0.673	–	–	–	–

The table is sorted by descending ranking orders of bias corrected efficiency scores of the MCH hospitals. G mean represents geometric mean. Refer to the Additional file 1: Appendix for full details

### Visual reporting of efficiency scores among the sample secondary MCH hospitals

**Fig. 1:**
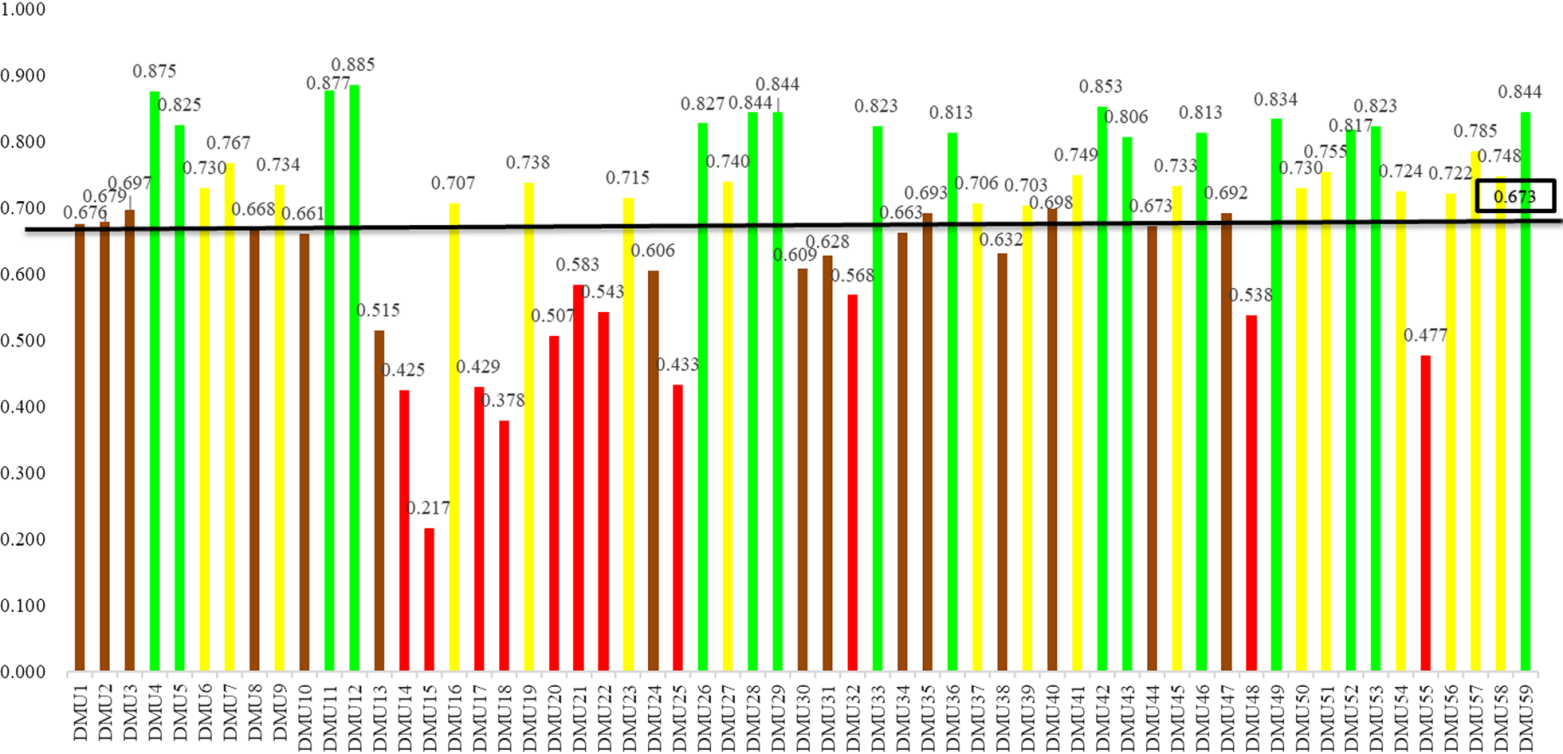
Distribution of bias-corrected efficiency scores of benchmarking among the secondary MCH hospitals

Figure 1 is a benchmarking of the bias-corrected efficiency scores among the 59 secondary MCH hospitals. Twenty secondary MCH hospitals had TE scores below the average (0.673). The TE distribution indicates that, none of the MCH hospitals fell into the excellent efficiency group; sixteen secondary MCH hospitals fell into the good efficiency group; seventeen secondary MCH hospitals fell into the average efficiency group, indicating ample room for improvement; fourteen secondary MCH hospitals fell into the poor efficiency group, representing the necessity to improve performance; and twelve secondary MCH hospitals fell into the failing efficiency group in which they need immediate improvement.

## Discussion

Our study found significant capacity variations among secondary MCH hospitals in terms of inputs and outputs indicators. For example, the maximum value of ‘number of equipment with value greater than 10,000 RMB Yuan' was 19.51 times than that of the minimum value, reflecting huge difference among secondary MCH hospitals in fixed assets as well as imbalance in resource allocation from the governments. The finding is consistent with Wang et al. (2016), who found distinct input disparities in health resource allocation in Heilongjiang Province, China [17]. Similar findings can be drawn on the output indicators. For example, the maximum value of ‘number of discharged patients’ was 297.97 times than that of the minimum value, suggesting the imbalance capacity development of the secondary MCH hospitals. Thus, it is suggested that the MCH hospitals should adjust inputs based on scientific evidences related to the input indicators for resource utilization, together with support from the government.

Because of incomplete data reporting by some DMUs, only 59 out of 74 secondary MCH hospitals in Hubei Province were qualified to apply DEA. Fifteen secondary MCH hospitals were found missing data on some of the key indicators, which reflected that the need to strengthen their information systems along with capacity building for better reporting. Moreover, many of the MCH hospitals in Hubei Province have installed different reporting systems from the one adopted by the government, while some county/district level MCH hospitals have yet to install the upgrading packages, resulting in the incompatibility of data transfer and the failure of data reporting in good quality. Therefore, it is necessary to further standardize data reporting with a uniform protocol, together with data quality control programs implemented for better reporting, such as promoting data flow between the information systems of the MCH hospitals and the information systems of the government, enabling data inter-connection and sharing among the information systems of different departments of the government [29].

In our study, we also found that the TE scores of 20 secondary MCH hospitals in Hubei Province were below average TE score. As TE can also be decomposed into pure technical efficiency (PTE) and scale efficiency (SE) [12], improvement activities and policies can primarily focus on improving PTE and SE respectively based on their actual situation. PTE can be improved in two directions. One is to improve internal management, and the other is to have policy interventions on environmental factors. In terms of internal management, secondary MCH hospitals can focus on the utilization of resources, structural and organizational factors such as leadership and governance, information system strengthening, capacity building, process optimization (layout of all hospital departments, implementation of clinical pathways, etc.), performance evaluation, while policy interventions can highlight the construction of health systems and create a suitable environment for the hospitals to play. SE means the MCH hospitals should scale up appropriately. Take DMUs 1 and 3, for example. Their TE scores were 1 before bias correction, but after bias correction, their ranking order were much lower than many other secondary MCH hospitals. Such finding can be interpreted in at least twofolds. First, the scales of the two hospitals measured by input indicators were much smaller than those of the other secondary MCH hospitals. Second, due to their geographic locations which are close to big hospitals, they face intense competition from them. Thus, secondary MCH hospitals need to not only make efforts to strengthen their capacity and scale, but also improve the quality of care so as to attract more patients to come for treatments, as a way to increase the TE.

According to international experience, performance evaluation can be used not only as a tool for internal improvement, but also as a governance tool to optimize resource allocation [9, 30]. In China, some general hospitals have introduced the methods of third-party performance evaluation in order to continuously improve their internal management [31]. However, in MCH hospitals, benchmarking management has yet to be applied for in-depth performance evaluation. It is suggested that the administration departments (both health administration and health insurance departments) commission a third-party performance evaluation agency to measure the TE and the overall performance of MCH hospitals regularly, as TE can only reflect one aspect of performance. Other dimensions, such as quality, cost, patient satisfaction, etc., should also be added [21]. The performance evaluation agency can use benchmarking management by identifying the MCH hospitals with best practices, setting them as a model for those with poor and failing performance to learn from. In addition, the performance evaluation agency can hold regular meetings and seminars based on the results, which can further be posted to the public. In this way, the efficiency and performance of the MCH hospitals can be monitored  over time, and both hospitals and government can make evidence-based decisions for better management and governance.

Our study has some strengths and limitations. As one of our first initiatives to apply Bootstrap-DEA to measure TE of secondary MCH hospitals in China, this study provided a direction to estimate more reliable efficiency scores compared with the application of classic DEA models. However, not all secondary MCH hospitals were included into our analysis due to incomplete data of some hospitals. Moreover, some indicators, like antenatal care and postnatal care visits are helpful indictors to depict MCH services in theory. However, these indicators data were collected by the hospitals and not reported to the health administration departments. Therefore, we were unable to include them into our analysis. This reflects the need for the health administration departments  to include these indicators into their monitoring list as well. Third, the decomposition of TE into PTE and SE was proposed by Fare (1994), while the Bootstrap-DEA was proposed by Simar & Wilson (1998). The FEAR package developed by Wilson (2001) does not support the decomposition of one year data. Therefore we can only discuss in theory how to improve the PTE and SE separately.

## Conclusions

Significant capacity variations existed among the secondary MCH hospitals in terms of input and output indicators and their overall TE was low in Hubei province of China. The secondary MCH hospitals in Hubei province have the potential to improve their TE, and our research framework helps generate a prioritized path for management and policy interventions. At the government level, health administration departments can provide policy support. At the secondary MCH hospital level, they can strengthen the information system construction of MCH hospitals and improve their internal management. Governments at every level are advised to explore third-party performance evaluation to improve efficiency and the overall performance of the secondary MCH hospitals. Moreover, the experience of Hubei province may have some policy implications for some LMICs to improve their MCH services.

## Supplementary Information

**Additional file 1: Table S2.** Efficiency scores and rankings before and after bias correction of MCH hospitals

## Data Availability

The data from which these findings were drawn is available from the corresponding author on reasonable request.

## Abbreviations

AE: Allocative efficiency
BCC: Banker, Charnes, and Cooper
CCR: Charnes, Cooper, and Rhodes
DEA: Data Envelopment Analysis
DMUs: Decision-making units
HCHBP: Health Commission of Hubei Province
MCH hospitals: Maternal and child health hospitals
SDGs: Sustainable Development Goals
SPSS: Statistical Product and Service Solutions
TE: Technical efficiency
WHO: World Health Organization
PTE: Pure technical efficiency
SE: Scale efficiency
LMICs: Low- and middle- income countries
