# Supplementary material for: The technical efficiency of maternal and child health hospitals in China: a case study of Hubei Province
Source: Reprod Health. 2022 Mar 31;19:85. doi: 10.1186/s12978-022-01386-x (PMC8973823; doi:10.1186/s12978-022-01386-x)
Supplement: Supplementary file 1 — Additional file 1: Table S2. Efficiency scores and rankings before and after bias correction of MCH hospitals [file 12978_2022_1386_MOESM1_ESM.docx]

**Appendix**

**Table 2** Efficiency scores and rankings before and after bias correction of MCH hospitals

| DMU | Before bias-correction | After bias-correction | Bias | Lower bound | Upper bound | Ranking orders（After(Before)） |
| --- | --- | --- | --- | --- | --- | --- |
| DMU 12 | 0.995 | 0.885 | 0.110 | 0.821 | 0.987 | 1(18) |
| DMU 11 | 0.977 | 0.877 | 0.101 | 0.828 | 0.970 | 2(20) |
| DMU 4 | 1.000 | 0.875 | 0.125 | 0.831 | 0.990 | 3(1) |
| DMU 42 | 0.971 | 0.853 | 0.118 | 0.789 | 0.965 | 4(21) |
| DMU 28 | 1.000 | 0.844 | 0.156 | 0.796 | 0.991 | 5(1) |
| DMU 59 | 1.000 | 0.844 | 0.156 | 0.801 | 0.990 | 6(1) |
| DMU 29 | 0.983 | 0.844 | 0.139 | 0.773 | 0.974 | 7(19) |
| DMU 49 | 1.000 | 0.834 | 0.166 | 0.797 | 0.991 | 8(1) |
| DMU 26 | 1.000 | 0.827 | 0.173 | 0.800 | 0.992 | 9(1) |
| DMU 5 | 0.935 | 0.825 | 0.110 | 0.760 | 0.927 | 10(23) |
| DMU 33 | 1.000 | 0.823 | 0.177 | 0.772 | 0.991 | 11(1) |
| DMU 53 | 1.000 | 0.823 | 0.177 | 0.786 | 0.992 | 12(1) |
| DMU 52 | 0.930 | 0.817 | 0.113 | 0.767 | 0.920 | 13(24) |
| DMU 36 | 0.946 | 0.813 | 0.133 | 0.737 | 0.942 | 14(22) |
| DMU 46 | 0.881 | 0.813 | 0.069 | 0.781 | 0.874 | 15(27) |
| DMU 43 | 0.925 | 0.806 | 0.119 | 0.755 | 0.917 | 16(25) |
| DMU 57 | 0.889 | 0.785 | 0.104 | 0.744 | 0.882 | 17(26) |
| DMU 7 | 0.825 | 0.767 | 0.058 | 0.730 | 0.818 | 18(31) |
| DMU 51 | 0.878 | 0.755 | 0.123 | 0.674 | 0.870 | 19(28) |
| DMU 41 | 0.834 | 0.749 | 0.085 | 0.708 | 0.827 | 20(29) |
| DMU 58 | 1.000 | 0.748 | 0.252 | 0.726 | 0.992 | 21(1) |
| DMU 27 | 1.000 | 0.740 | 0.260 | 0.705 | 0.990 | 22(1) |
| DMU 19 | 1.000 | 0.738 | 0.262 | 0.720 | 0.989 | 23(1) |
| DMU 9 | 1.000 | 0.734 | 0.266 | 0.717 | 0.990 | 24(1) |
| DMU 45 | 1.000 | 0.733 | 0.267 | 0.680 | 0.989 | 25(1) |
| DMU 6 | 1.000 | 0.730 | 0.270 | 0.711 | 0.990 | 26(1) |
| DMU 50 | 1.000 | 0.730 | 0.270 | 0.711 | 0.991 | 27(1) |
| DMU 54 | 0.815 | 0.724 | 0.091 | 0.680 | 0.809 | 28(32) |
| DMU 56 | 0.771 | 0.722 | 0.050 | 0.696 | 0.764 | 29(38) |
| DMU 23 | 0.780 | 0.715 | 0.065 | 0.688 | 0.773 | 30(36) |
| DMU 16 | 0.778 | 0.707 | 0.071 | 0.663 | 0.771 | 31(37) |
| DMU 37 | 0.791 | 0.706 | 0.085 | 0.667 | 0.784 | 32(33) |
| DMU 39 | 0.826 | 0.703 | 0.123 | 0.641 | 0.819 | 33(30) |
| DMU 40 | 0.762 | 0.698 | 0.064 | 0.671 | 0.755 | 34(40) |
| DMU 3 | 1.000 | 0.697 | 0.303 | 0.667 | 0.991 | 35(1) |
| DMU 35 | 0.786 | 0.693 | 0.093 | 0.649 | 0.779 | 36(34) |
| DMU 47 | 0.783 | 0.692 | 0.091 | 0.655 | 0.778 | 37(35) |
| DMU 2 | 0.768 | 0.679 | 0.089 | 0.646 | 0.761 | 38(39) |
| DMU 1 | 1.000 | 0.676 | 0.324 | 0.655 | 0.992 | 39(1) |
| DMU 44 | 1.000 | 0.673 | 0.327 | 0.648 | 0.992 | 40(1) |
| DMU 8 | 0.711 | 0.668 | 0.043 | 0.636 | 0.706 | 41(45) |
| DMU 34 | 0.756 | 0.663 | 0.093 | 0.627 | 0.749 | 42(41) |
| DMU 10 | 0.722 | 0.661 | 0.061 | 0.627 | 0.716 | 43(43) |
| DMU 38 | 0.721 | 0.632 | 0.089 | 0.579 | 0.714 | 44(44) |
| DMU 31 | 0.736 | 0.628 | 0.107 | 0.574 | 0.730 | 45(42) |
| DMU 30 | 0.692 | 0.609 | 0.084 | 0.576 | 0.685 | 46(46) |
| DMU 24 | 0.689 | 0.606 | 0.084 | 0.572 | 0.683 | 47(47) |
| DMU 21 | 0.620 | 0.583 | 0.037 | 0.559 | 0.615 | 48(51) |
| DMU 32 | 0.659 | 0.568 | 0.091 | 0.534 | 0.655 | 49(48) |
| DMU 22 | 0.625 | 0.543 | 0.081 | 0.510 | 0.620 | 50(49) |
| DMU 48 | 0.622 | 0.538 | 0.084 | 0.503 | 0.616 | 51(50) |
| DMU 13 | 0.602 | 0.515 | 0.087 | 0.466 | 0.597 | 52(52) |
| DMU 20 | 0.547 | 0.507 | 0.040 | 0.482 | 0.543 | 53(54) |
| DMU 55 | 0.557 | 0.477 | 0.081 | 0.430 | 0.553 | 54(53) |
| DMU 25 | 0.464 | 0.433 | 0.032 | 0.415 | 0.460 | 55(56) |
| DMU 17 | 0.464 | 0.429 | 0.034 | 0.409 | 0.459 | 56(57) |
| DMU 14 | 0.469 | 0.425 | 0.044 | 0.403 | 0.465 | 57(55) |
| DMU 18 | 0.419 | 0.378 | 0.040 | 0.360 | 0.414 | 58(58) |
| DMU 15 | 0.239 | 0.217 | 0.022 | 0.207 | 0.236 | 59(59) |
| G mean | 0.789 | 0.673 | - | - | - | - |

Note*: The table is sorted by descending ranking orders of bias corrected efficiency scores of the MCH hospitals. G mean represents geometric mean. Refer to the Appendix for full details.
